# Ectopic germinal centers in the thymus accurately predict prognosis of myasthenia gravis after thymectomy

**DOI:** 10.1038/s41379-022-01070-2

**Published:** 2022-03-25

**Authors:** Joona Sarkkinen, Johannes Dunkel, Anni Tuulasvaara, Antti Huuskonen, Sari Atula, Eliisa Kekäläinen, Sini M. Laakso

**Affiliations:** 1 Translational Immunology Research Program, University of Helsinki, Helsinki, Finland; 2 Department of Pathology, University of Helsinki and Helsinki University Hospital, Helsinki, Finland; 3 Department of Neurology, Helsinki University Hospital, Helsinki, Finland; 4 Department of Pediatric Cardiac and Transplantation Surgery, The New Children’s Hospital, University of Helsinki and Helsinki University Hospital, Helsinki, Finland; 5 HUS Diagnostic Center, HUSLAB, Clinical Microbiology, Helsinki, Finland

**Keywords:** Thymus, Neuroimmunology, Autoimmunity

## Abstract

The ability of thymic histopathology to predict the long-term impact of thymectomy in non-thymomatous myasthenia gravis (NTMG) is mainly uncharted. We applied digital pathology to quantitatively characterize differences of thymic histology between early-onset (EOMG) and late-onset MG (LOMG) and to investigate the role of thymic changes for thymectomy outcomes in MG. We analyzed 83 thymic H&E slides from thymectomized NTMG patients, of which 69 had EOMG and 14 LOMG, using digital pathology open-access software QuPath. We compared the results to the retrospectively assessed clinical outcome at two years after thymectomy and at the last follow-up visit where complete stable remission and minimal use of medication were primary outcomes. The automated annotation pipeline was an effective and reliable way to analyze thymic H&E samples compared to manual annotation with mean intraclass correlation of 0.80. The ratio of thymic tissue to stroma and fat was increased in EOMG compared to LOMG (*p* = 8.7e-07), whereas no difference was observed in the ratio of medulla to cortex between these subtypes. AChRAb seropositivity correlated with the number of ectopic germinal centers (eGC; *p* = 0.00067) but not with other histological areas. Patients with an increased number of eGCs had better post-thymectomy outcomes at two years after thymectomy (*p* = 0.0035) and at the last follow-up (*p* = 0.0267). ROC analysis showed that eGC area predicts thymectomy outcome in EOMG with an AUC of 0.79. Digital pathology can thus help in providing a predictive tool to the clinician, the eGC number, to guide the post-thymectomy treatment decisions in EOMG patients.

## Introduction

Myasthenia gravis (MG) is a rare neuroimmunological disease characterized by exercise-induced muscle weakness with an overall prevalence of 60–100 cases per million^1^. Ten percent of MG patients have symptoms limited to the ocular muscles, while the majority have a generalized disease^2^. The disease is caused by autoantibodies targeting proteins of the neuromuscular junction; in 85% of patients with generalized MG these autoantibodies are against acetylcholine receptor (AChR) and in 5% against muscle-specific kinase^3^.

The thymus has a central role in MG. Thymomatous MG (TAMG) is however a rare finding, diagnosed in only 10–15% of MG patients, and the majority of MG patients have non-thymomatous MG (NTMG). NTMG is divided into two main subgroups based on age at disease onset: early-onset MG (EOMG) starting before the age of 50 years, and late-onset MG (LOMG) starting thereafter. Of EOMG patients, 60% are estimated to have thymic hyperplasia, whereas hyperplasia is seen in only 10–15% of LOMG patients in which thymic involution represents the dominant finding^4,5^. Ectopic germinal centers (eGC), which are thought to develop after chronic local inflammation^6^, are one of the most prominent findings in the thymi of MG patients and they are found in most EOMG patients, but only in less than 25% of LOMG patients^7^. Especially in EOMG, AChR antibodies (AChRAbs) are produced in the thymus^8^ and the amount of eGCs and thymic hyperplasia correlates with AChRAb titer^7^, emphasizing the significance of thymic pathology for the development of MG.

Hence, it is not surprising that thymectomy is often the cornerstone of the treatment of generalized MG in addition to ACh-esterase inhibitors (AChEi) and immunosuppressants. In a randomized trial of 126 patients, Wolfe et al. found thymectomy plus prednisone superior to prednisone alone in a follow up of up to 5 years in AChRAb positive NTMG with generalized symptoms in both patients under and over 40 years^9,10^. However, in patients over 50 years of age, there were no statistical differences between the subgroups in post-hoc analysis. In a systematic review and meta-analysis by Zhang et al., thymectomy was not superior to conservative treatment measured by complete stable remission or pharmacological remission in LOMG^11^. According to current guidelines, in AChRAb positive NTMG with generalized symptoms, thymectomy should be considered especially for patients under 50 years^12^.

Besides age at onset and thymic hyperplasia, there are currently no other clinical variables which would predict the effect of thymectomy in generalized AChRAb positive NTMG^13–15^. Most patients do not reach remission after operative treatment, and the use of immunosuppressants is often advised for years, which can lead to severe side effects. Better biomarkers to identify patients with a high likelihood of remission are thus needed to guide treatment decisions.

The significance of thymic histopathology in estimating long-term impact of thymectomy in NTMG is mainly unknown. Thymic histopathology is typically assessed manually from hematoxylin-eosin (H&E) sections without thorough quantitative analysis if there are no clinical suspicions of thymoma nor signs of thymoma or carcinoma in H&E stained thymic sections. The objective of this study was to investigate NTMG thymi using modern tools of digital pathology and to compare pathological findings with clinical information to predict postoperative prognosis.

## Materials and methods

### Study setting

**Fig. 1:**
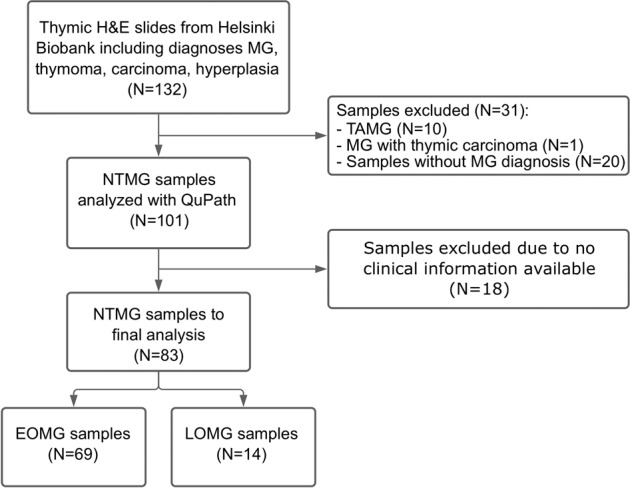
Flow diagram showing the selection of the clinical cohort. In total, 132 thymic HE slides were available from Helsinki Biobank after request, of which 31 were excluded due to thymic malignancy or a diagnosis other than MG. After the analysis of 101 NTMG samples using QuPath, 18 samples were excluded from further analysis because clinical follow up data was missing. H&E, hematoxylin and eosin; MG, myasthenia gravis; NTMG, non-thymomatous myasthenia gravis.

This study complies with the STARD reporting guidelines for diagnostic testing and was performed in accordance with the Declaration of Helsinki under the ethical approval (HUS/747/2019) granted by Helsinki University Hospital Ethical Review Board^16^. From Helsinki Biobank we requested all formalin-fixed paraffin embedded (FFPE) thymus samples with the diagnoses according to ICD-10 classification containing MG (G70.0), thymoma (C37), carcinoma (C37), and hyperplasia (E32.0). This gave us a sample cohort (*N* = 132) of thymus samples from thymectomies performed in Helsinki University Hospital between years 1980 and 2019. All samples had been initially clinically reviewed by a pathologist at the time of thymectomy. The single most representative H&E stained thymic section from every sample was retrospectively digitized by Helsinki Biobank using Pannoramic 250 Flash III scanner (3DHISTECH, Budapest, Hungary) and 20X objective. Digitized sections and histopathological diagnoses were then re-evaluated by a pathologist (J.D.) and compared to original diagnoses in the patient records. MG diagnoses were confirmed from the patient records of Helsinki University Hospital. Samples with TAMG or MG with thymic carcinoma (*N* = 11), and samples without MG diagnosis (*N* = 20) based on review of patient records, were excluded from the analysis. In total, *N* = 101 thymic H&E sections were accepted for further analysis (Fig. 1).

In addition, we collected thymus samples from 37 pediatric patients, of which 21 were females (56.8%), undergoing corrective open-heart surgery between the ages of 4 days and 6 years (ethical approval HUS/747/2019). H&E stained FFPE sections from these samples were used as healthy controls to compare histology of EOMG and LOMG thymi.

### Clinical characteristics

The clinical data of the patients who underwent thymectomy were retrospectively collected from the patient records of Helsinki University Hospital. Clinical data were available from 83 of 101 NTMG patients. Diagnosis of MG was made by the attending neurologist based on clinical examination, AChRAb measurement, repetitive nerve stimulation and single-fibre electroneuromyography, according to the current guidelines^17^. After thymectomy, patients were followed up in the Department of Neurology of Helsinki University Hospital or by the neurology department of the referring hospital. Postoperative outcome at two years after thymectomy was evaluated according to need of AChEi and immunosuppressant medication compared to preoperative state and classified into three groups: (i) pharmacological remission (PR), (ii) improved compared to preoperative state, and (iii) unchanged compared to preoperative state. PR was defined as the patient not receiving AChEi or immunosuppressive medications at two years after thymectomy. Improved was defined as the patient having 50% reduction in the amount of AchEi in use compared to preoperative state or no immunosuppressive medications in use. Postoperative outcome at the last follow-up visit was classified into three groups: (I) minimal need of medication (MM), (II) moderate response, and (III) no response. MM was defined as the patient receiving no or low dose of AChEi (≤100 mg/day) and not using immunosuppressants during the last year. Moderate response was defined as the patient receiving AChEi with a dose of over 100 mg/day and/or immunosuppressants, but where no myasthenic crisis had occurred during follow-up after thymectomy. No response was defined as the patient having a myasthenic crisis during follow-up after thymectomy, which required treatment in an intensive care unit or giving high dose intravenous methylprednisolone, performing plasmapheresis, or administering intravenous immunoglobulin.

### Digital pathology

**Fig. 2:**
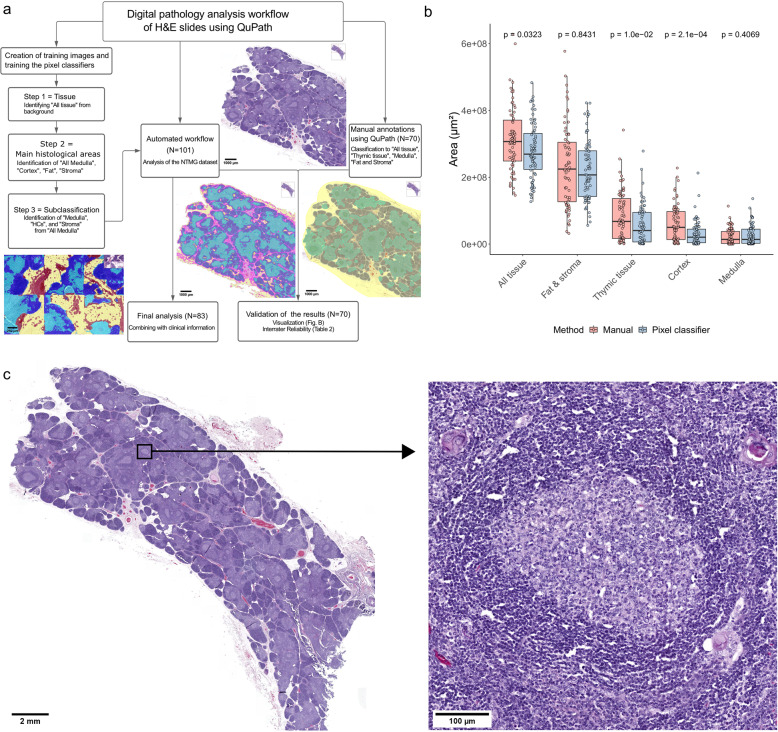
Automated digital pathology analysis using QuPath. **a** Flow diagram illustrating the digital pathology analysis workflow using QuPath. **b** Boxplot comparison between annotated area per sample and histological areas using manual annotation (dots) and automated pixel classifier-based workflow (triangles). “All tissue” refers to all tissue per sample and “Thymic tissue” to summation of medulla and cortex. *P* values were calculated using the unpaired Wilcoxon test: * = 0.032, ** = 0.01, *** = 0.00021, ns = non-significant. **c** A typical H&E image from MG patient with thymic hyperplasia with several ectopic germinal centers. Images were obtained from CaseViewer using x 0.7 (left) and x 20 (right) objectives.

Digitized H&E thymic slides from MG patients and pediatric controls were investigated using digital pathology image analysis open-source software QuPath^18^. For the analysis, we trained three-pixel classifiers which all used Random trees as an algorithm. The first step of the analysis identified the tissue (“All tissue”) from the background. The second step classified tissue to “All Medulla”, “Cortex”, “Fat”, and “Stroma” followed by the third step, a subclassification of the “All Medulla” further to “Medulla”, “Stroma” and “Hassall’s Corpuscles”, after which data was exported from the QuPath as.csv files. Training of pixel classifiers was done using training images containing different tissue regions from ten different thymic samples from a dataset of *N* = 101 NTMG samples. Flow diagram of the analysis pipeline with example images is shown in Fig. 2a.

To validate performance of the pixel classifier, J.S. and pathologist J.D. together manually annotated 70 thymic samples from a data set of *N* = 101 NTMG samples using QuPath. Interrater reliability (IRR) for annotated histological areas between pixel classifiers and manual annotation were calculated and are shown in results (Table 2). eGCs were annotated manually by the pathologist J.D. and A.T. using QuPaths wand tool since intensities of eGCs were similar with cortex, and thus, it would not have been feasible to use the pixel classifiers. Mantle zones of lymphatic follicles were not included in annotations. An example of H&E stained thymic slide from an MG patient exhibiting thymic hyperplasia and several eGCs is shown in Fig. 2c. Images were obtained from CaseViewer (3DHISTECH, Budapest, Hungary) using ×0.7 (left) and ×20 (right) objectives.

### Statistical analysis

Figures and statistics were done in R Studio using packages dplyr, ggplot2 gridExtra, irr, ggpubr, and ROCR^19^. H&E images were edited for figures using FiJI^20^ and flow diagrams were created in Lucidchart (www.lucidchart.com). Descriptive statistics for all variables were performed. Non-parametric statistical tests were performed to compare the relationship between a quantitative variable among different categories of another variable. Unpaired Wilcoxon test was used to compare two different categories and Kruskal–Wallis test was used to compare multiple categories. Intra-observer agreement for different areas of different histological regions between pathologists’ and automated workflow was analyzed using the Intraclass Correlation Coefficient (ICC). Correlation plot analysis was performed using Spearman’s correlation test. Receiver Operating Characteristics (ROC) curve analysis was performed to evaluate the performances of the generalized linear models to predict the outcome after thymectomy. *P* values of <0.05 were considered statistically significant.

## Results

### EOMG and LOMG patients of the study cohort show common clinical features

A total of 83 NTMG patients were accepted for final analysis, and the characteristics of the patients are shown in Table 1. A total of 69 patients (83%) had EOMG and 14 (17%) LOMG, of which 63 (91%) and 4 (29%) were women, respectively. Seventy-five patients (90%) had generalized MG at the time of operation and 8 (10%) had ocular disease. Proportion of AChRAb positive patients did not differ between EOMG and LOMG (68% vs 71%). Thymic histopathology was statistically different between subgroups; thymic hyperplasia was detected in 74% of EOMG and 14% of LOMG patients (*p* = 0.001). Involuted thymus was considered as normal histology of the aging thymus. Mean follow-up time after thymectomy was 15 years (SD ± 9.2 y) for EOMG and 10 y (SD ± 4.7 y) for LOMG patients. There were no significant differences between EOMG and LOMG patients’ outcomes at two years follow up.

**Table 1:** Patient characteristics.

	EOMG (N = 69)	LOMG (N = 14)	P-value
Gender			
Female	63 (91%)	4 (29%)	<0.001
Male	6 (9%)	10 (71%)	
Age at diagnosis			
Mean (SD)	32 (±10)	62 (±8.9)	
Age at thymectomy			
Mean (SD)	33 (±11)	64 (±8.4)	<0.001
AChRAb			
Negative	14 (20%)	3 (21%)	0.992
Positive	47 (68%)	10 (71%)	
Missing	8 (11.6%)	1 (7.1%)	
Thymic pathology			
Normal	18 (26%)	12 (86%)	<0.001
Hyperplasia	51 (74%)	2 (14%)	
Ocular vs generalized			
Ocular	6 (9%)	2 (14%)	0.521
Generalized	63 (91%)	12 (86%)	
Outcome after two years			
i) PR	11 (16%)	2 (14%)	0.647
ii) Improved	14 (20%)	4 (29%)	
iii) Unchanged	42 (61%)	7 (50%)	
Missing	2 (2.9%)	1 (7.1%)	
Outcome at last follow-up			
I) MM	19 (28%)	4 (29%)	0.949
II) Moderate	27 (39%)	5 (36%)	
III) No effect	20 (29%)	4 (29%)	
Missing	3 (4.3%)	1 (7.1%)	
Follow up time in years			
Mean (SD)	15 (±9.2)	10 (±4.7)	0.0937
Missing	4 (5.8%)	1 (7.1%)	

*P* values were calculated using either the unpaired Wilcoxon test (numeric variables) or the Kruskal-Wallis test (categorical variables). PR pharmacological remission; Improved, −50% reduction in AChEi dose or no immunosuppressants in use; Unchanged, no change compared to preoperative state; MM minimal need of medication, no or low dose AChEi (≤100 mg/day) and no immunosuppressants; Moderate, AChEi dose over 100 mg/day or use of immunosuppressants; No effect, myasthenic crisis within one year.

### Digital pathology analysis using QuPath is reliable and effective

Analysis of H&E slides with automated workflow using QuPath performed similarly to manual annotation (Fig. 2a, b). The ICC values for “All tissue”, “Thymic tissue”, “Medulla”, “Cortex”, “Fat and stroma” were 0.845 (CI95% 0.55–0.931), 0.877 (CI95% 0.218–0.961), 0.602 (CI95% 0.147–0.802), 0.736 (CI95% 0.736–0.901), and 0.819 (CI95 0.725–0.884), respectively (Table 2).

**Table 2:** The Intraclass Correlation Coefficients (ICCs) of histological areas between two annotation methods, manual annotation and automated annotation using pixel classifiers.

Area (µm²)	ICC	ICC 95% CI	*P* value
All	0.845	[0.55, 0.931]	9.35E-05
Thymic tissue	0.877	[0.218, 0.961]	0.0074
Cortex	0.602	[0.147, 0.802]	0.00572
Medulla	0.837	[0.736, 0.901]	5.98E-16
Stroma + Fat	0.819	[0.725, 0.884]	6.99E-19

On average, the pixel classifiers of automated workflow annotated the tissue areas stricter and hence found less tissue in every forementioned histological areas. The differences between manual annotation and automated classifications were statistically significant in “All tissue” (*p* = 0.045), “Thymic tissue” (*p* = 0.012), and “Cortex” (*p* = 0.0003) (Fig. 2b). Of note, different performances in “Cortex” could have been affected by red blood cells on cortical areas in a minority of samples, which machine learning-based pixel classifiers classified as stroma and the pathologist classified as cortex based on the ground truth.

### Thymus histology in EOMG is different from LOMG

**Fig. 3:**
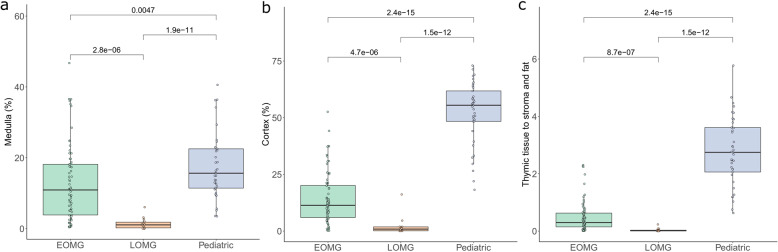
Thymic tissue is more prevalent in EOMG than LOMG. Area of medulla and cortex are shown as percentages of all tissue per sample. The ratio of thymic tissue to the combined area of stroma and fat is also shown. *P* values were calculated using Kruskal-Wallis test.

Total tissue area (“All tissue”) annotated per thymic section did not differ between the EOMG and LOMG groups. Pediatric samples had significantly smaller amounts of tissue in thymic sections than EOMG (*p* < 2.22e-16) and LOMG (*p* = 1.5e-12; Fig. S1A). These results from the smaller resection size and the different surgical techniques used to obtain the samples, i.e., MG thymi were collected from total thymectomies, while pediatric open heart surgery includes a partial thymectomy. Other histological areas are shown as percentages of total tissue per thymic section. As earlier shown with a more qualitative assay^21^, the quantitative digital pathology analysis confirmed that EOMG patients had more thymic (*p* = 8.7e-07), medullar (*p* = 2.8e-06), cortical (*p* = 4.7–06) and stromal tissue (*p* = 0.0094) and less fat (1.6e-06) compared to LOMG patients. The ratio of thymic tissue (medulla + cortex) to stroma and fat was 11 times lower in LOMG than EOMG thymi (*p* = 8.7e-07) which can be the result of normal age-related involution of the thymus (Fig. 3 and Fig. S1A). Medulla to cortex ratio did not differ between MG subtypes. Above mentioned main histological areas did not correlate with the outcomes after thymectomy at two years or at the last follow up visit (Fig. S1B, C).

In pediatric control samples, the ratio of thymic tissue to stroma and fat was higher than in EOMG and LOMG samples. There was no statistical difference between pediatric and EOMG thymi in the percentage of medullary area (Fig. S1D).

Since the number of Hassall Corpuscles (HC) per area of thymic medulla have been reported to be increased in MG patients with thymic hyperplasia^22^, we next evaluated using our pixel classifiers whether area of HCs increased independently from medullary tissue. Area of HCs was larger on average in all MG thymi compared to pediatric samples (*p* = 8.4e-07) and the difference was more pronounced when we compared only EOMG patients to pediatric samples (*p* = 5.9e-08; Fig. S2A). MG patients with normal thymus or with thymic hyperplasia had more HCs compared to pediatric samples (*p* = 0.012 and *p* = 4.1e-8), but there was no statistical difference in HCs between MG patients with normal thymus or with thymic hyperplasia (*p* = 0.059, Fig. S2B). Interestingly, female MG patients had more HCs than their male counterparts (*p* = 0.0052), a difference which could not be detected in pediatric samples (Fig. S2C).

### AChRAb status correlates with area of eGCs

Thymic samples from AChRAb seronegative patients compared to seropositive patients did not show significant differences in percentages of medulla, stroma, or fat of the total tissue area nor in ratios of thymic tissue to stroma and fat, medulla to cortex, or HCs to medulla (Fig. S3A). However, as reported by Truffault et al and reproduced here, AChRAb seropositive patients had more eGCs in the thymus than seronegative patients (Fig. S3A)^7^. Subgroup analysis revealed that this correlation could be found only in EOMG but not in LOMG thymi. In EOMG samples, seropositive patients had also higher proportion of cortex of the total tissue than seronegative patients (*p* = 0.043), whereas in LOMG samples, seronegative patients had slightly higher proportion of cortex than the seropositive patients (*p* = 0.011; Fig. S3B). Other histological areas did not differ between AChRAb seronegative or positive samples.

### Ectopic germinal centers are more prevalent in EOMG thymi

The presence of thymic eGCs in MG is well known, and in our sample set, 70% of all MG patients, 73% of EOMG patients, and 57% of LOMG had at least one eGC per thymic section, which is in line with earlier reports^23^. EOMG patients had 4.8 times more eGCs and 11 times larger eGC area per thymic section than LOMG patients (*p* = 0.025 and *p* = 0.012). Patient’s age at the time of thymectomy correlated inversely with the prevalence of eGCs; after the age of 45 years the number of eGCs per thymic section was negligible (Fig. S4A). In MG patients over 45 years of age at the time of thymectomy, the median number of eGCs was zero (IQR 0.0–3.0), with only one patient with a maximum of seven eGCs per thymic section. In contrast, in patients under 45 years, the median number of eGCs was 5.5 (IQR 1.0–15.0).

### High number of ectopic germinal centers in the thymus predicts better clinical outcome after thymectomy in EOMG patients

**Fig. 4:**
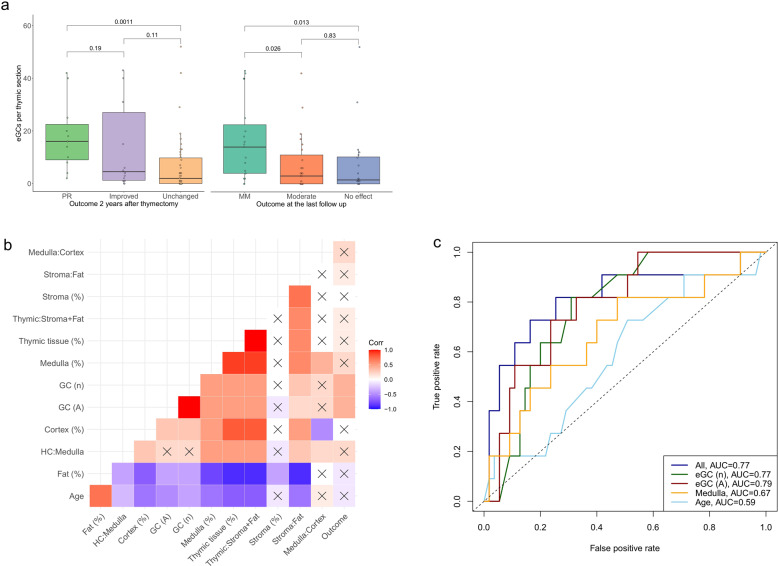
Number and area of thymic eGCs predict outcome after thymectomy in EOMG. **a** Outcome two years after thymectomy. PR, pharmacological remission; Improved, −50% reduction in AChEi dose or no immunosuppressants in use; Unchanged, no change compared to preoperative state. Outcome at the last follow-up. MM, minimal need of medication, no or low dose AChEi (≤100 mg/day) and no immunosuppressants; Moderate, AChEi dose over 100 mg/day or use of immunosuppressants; No effect, myasthenic crisis within one year. Outcome measures are explained in more detail in materials and methods. *P* values were calculated using the Kruskal-Wallis test. **b** Correlation plot demonstrating the correlation between histopathologic areas, age, and 2-year outcome. Age, age at operation; %, percentage of all tissue; ratio between histological areas, Outcome, outcome at 2 years as a dummy variable where groups ii and iii were pooled together; A, area; n, number. *P* values were calculated using the Spearman’s correlation test and correlations with *p* values ≥ 0.05 are crossed over. **c** ROC curve of performance of generalized linear models to predict outcome at 2 years after thymectomy. All, all histological areas, their ratios, and age at operation.

Comparing the number and area of eGCs to the outcome after thymectomy revealed that eGCs correlate with better outcome both at two years after thymectomy and at the last follow up. EOMG patients who achieved PR 2 years after thymectomy had more eGCs (*p* = 0.0011) than EOMG patients whose condition was unchanged (Fig. 4a). Number of eGCs in the removed thymus also correlated with the outcome at the last follow up: patients in minimal need of medication (MM) group had more eGCs than patients with moderate outcome (*p* = 0.026) and patients with no clinical response (*p* = 0.013; Fig. 4a). Similarly, eGC area also differed between outcome subgroups in EOMG patients: eGC area was larger in patients with pharmacological remission at 2 years after thymectomy than in unchanged group (*p* = 0.0007). Also, the patients with minimal need of medication at the last follow-up had higher eGC area than the patients in moderate response group (*p* = 0.015) and in the no response group (*p* = 0.027; Fig. S4B). There was no correlation in LOMG patients between eGCs and outcome (Fig. S4C).

Using variables obtained from digital pathology analysis, we tested whether we could predict the outcome at two years after thymectomy in EOMG patients. A correlation plot where improved and unchanged groups were pooled together showed that only eGC number and area reached a statistically significant correlation with the outcome (Fig. 4b). The correlation plot summarizes the dependencies illustrated in Figs. 3 and S1A, B. Next, we created generalized linear models to predict outcome groups where improved and unchanged groups were pooled together. Performances of models were tested using ROC curves (Fig. 4c) and the area under the curves (AUCs) were determined. Interestingly, models based on only eGCs’ total area (AUC = 0.79) or number (AUC = 0.77) performed equally to using all histopathological areas (AUC = 0.77), which indicated that the predictive power of eGCs was superior to that of other histopathological areas. In comparison, a model using the area of medulla performed significantly weaker (AUC = 0.67). Models based on the ratio of thymic tissue to stroma and fat, which is an indicator of thymic hyperplasia, or patient’s age at thymectomy were not predictive (AUC = 0.59 in both).

## Discussion

To our knowledge, this is the first study to perform a thorough digital pathology analysis to systematically investigate histopathological changes in MG thymi. Our results show that automated annotation using QuPath is a reliable method to assess histology of the thymus from basic H&E staining. Here, we confirmed that the ratio of medulla and cortex to stroma and fat is increased in EOMG compared to LOMG^21^. No statistical difference was observed between disease subtypes in ratio of medulla to cortex, suggesting that hyperplastic changes affect the thymus as a whole and not specifically the medulla or cortex. AChRAb seropositivity was correlated with the number of eGCs in EOMG but not in LOMG, which supports the earlier findings that AChRAbs would derive from the thymic eGCs in EOMG^8^. Importantly, our results showed that a higher number of thymic eGCs was correlated with better postoperative outcome both at two years after thymectomy and at the last follow-up, and that eGCs are a potent predictor of post-thymectomy outcome in EOMG.

Based on our data, HCs are increased in EOMG patients compared to pediatric samples, but the difference between EOMG and LOMG patients or LOMG patients and pediatric samples was not statistically significant. However, our results agree with Matsui et al showing a trend of more HCs in MG with thymic hyperplasia than MG with normal histology of the thymus^22^. According to the correlation plot, HCs were among the few variables correlating with better post-thymectomy outcomes, even though this did not reach statistical significance. It should be noted that staining variation and calcification of HCs may hinder the performance of the pixel classifier based on H&E staining, and the significance of HCs in MG should be further investigated with immunohistochemistry and a larger sample size.

A clear limitation of our study is its retrospective nature. Our sample cohort contains several biases of choice, starting from the surgical method used for thymectomy. Only one thymic H&E slide per patient was included in our study, hence it is possible that our results do not reflect the overall thymic histological profile of MG. On the other hand, the current study underlines the vast potential of biobank research and digital pathology applied to archival samples. For a rare disease such as MG, our sample set is already substantial, although the small number of LOMG patients limits proper subgroup analysis between EOMG and LOMG.

Contrary to an earlier report^15^, we found that the number of thymic eGCs correlated with better post-thymectomy outcome. The discrepancy between our findings and the findings by Moran et al. might be caused by smaller sample size used in their study and differences in analysis. Even though our findings underline the role of eGCs to the pathogenesis of MG, their origin is still an enigma. MG thymus is reported to have signs of chronic viral infection, especially caused by Epstein-Barr virus, which could lead to chronic inflammation and thus predispose to eGCs and to MG, but the findings of Epstein-Barr virus infection in MG are contradictory^6,24–26^. As shown here and by Truffault et al, AChRAb seropositive EOMG patients have an increased number of thymic eGCs compared to their seronegative counterparts. Frequency of eGCs correlates also with higher AChRAb titers^7^. We could not detect a similar correlation in LOMG, and this suggests that LOMG AChRAbs could originate from outside of the thymus at least in patients with thymic involution. The number of eGCs declines with age, and patients over 45 years of age have similar thymic eGC numbers as LOMG patients^7^. The cut-off between EOMG and LOMG has long been debated. Our results showed that the number of eGCs decreased drastically after the age of 40 years and after the age of 45 years the number of eGCs was negligible. With the findings in our study and previous studies of eGC number correlating with key clinical features of EOMG, such as thymic antibody production and response to thymectomy, the age of 45 years could be thus a better biological cut-off for EOMG and LOMG than the currently used age of 50 years^7,27^.

In our sample set, with only a few exceptions, patients who reached minimal need of medication were all under 40 years at the MG diagnosis. Although increased thymic eGC frequency correlated with better post-thymectomy outcome, some EOMG patients who did not benefit from thymectomy had high numbers of eGCs. Recent findings suggest that pathogenic B cells can escape the thymus and affect treatment responses especially in MG patients who do not benefit from thymectomy^28^. Thus, better means to identify patients with a worse prognosis after thymectomy is needed, and subsequent early immunotherapies should be assessed based on individual biomarkers.

Here, we show that a high number of ectopic germinal centers in MG thymus at the time of thymectomy correlates with a better post-thymectomy outcome, and that lack of thymic ectopic germinal centers could be used as a predictive marker for disease progression in EOMG. Ectopic germinal centers can be identified and counted by an experienced histopathologist in routine H&E stained sections, with concurrent immunohistochemistry for germinal center markers (e.g. CD10 or BCL6), as necessary. Such a semi-quantitative scoring method could be more practical in routine diagnostic settings even though automated diagnostic pipelines to assess ectopic germinal centers should be developed in the future. Digital pathology can thus help the pathologist to provide a key parameter on the likely effect of thymectomy on EOMG patients.

## Supplementary information

Supplemental material

## Data Availability

The datasets used and analyzed during the current study are available from the corresponding author on reasonable request.
