# Supplementary material for: Ectopic germinal centers in the thymus accurately predict prognosis of myasthenia gravis after thymectomy
Source: Mod Pathol. 2022 Mar 25;35(9):1168–74. doi: 10.1038/s41379-022-01070-2 (PMC9424113; doi:10.1038/s41379-022-01070-2)
Supplement: Supplementary file 1 — Supplemental material [file 41379_2022_1070_MOESM1_ESM.pdf]

# Supplemental material

Summary of supplementary files

Fig. S1. Boxplots of main histological areas.

Fig. S2. Comparison of area of Hassall Corpuscles (HCs) between EOMG, LOMG and healthy pediatric thymi.

Fig. S3. Boxplots of AChRAb status and main histological areas.

Fig. S4. eGCs are more common in young MG patients and their prevalence is correlated with better outcome after thymectomy.

# Supplemental Figure 1

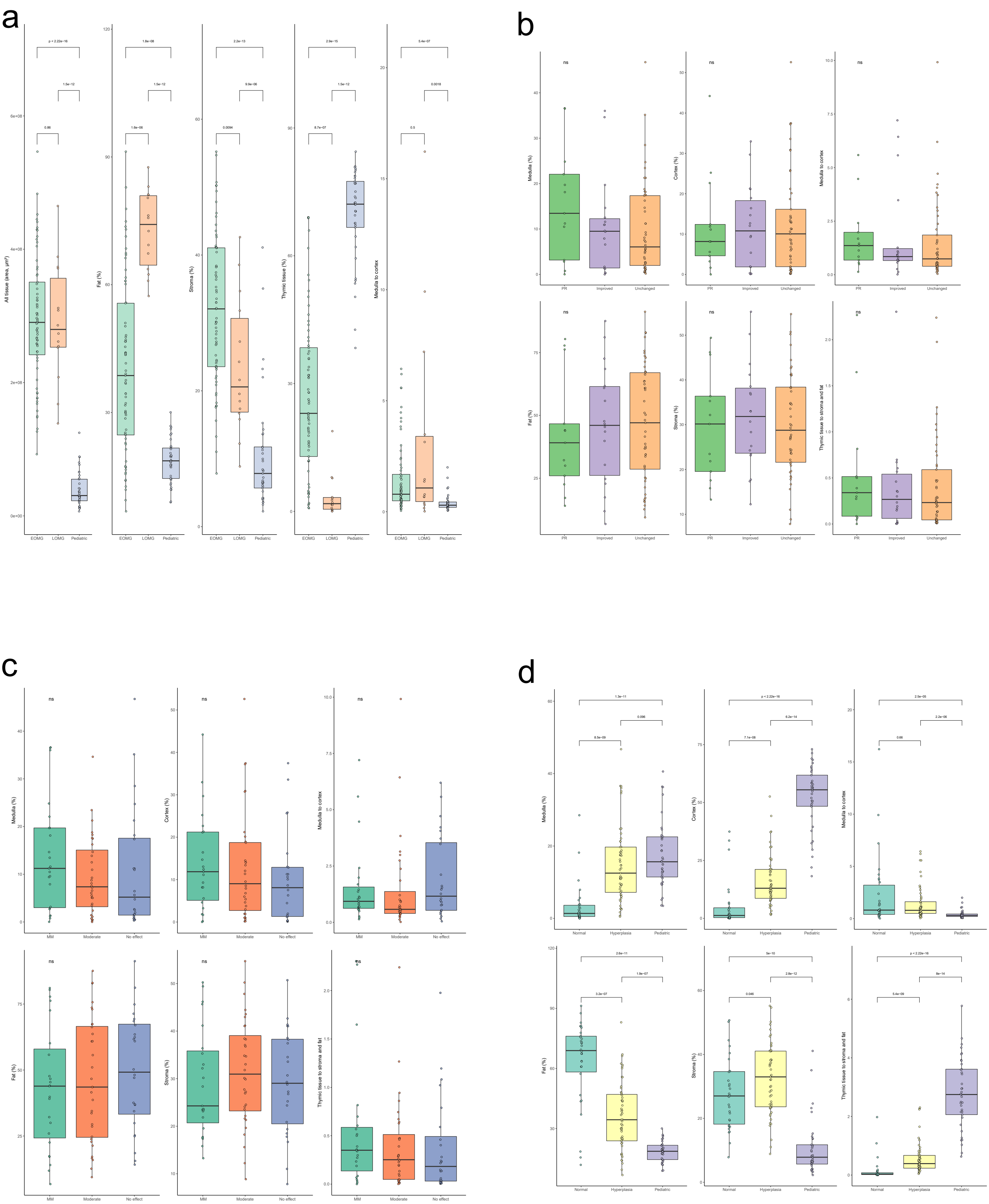


**Fig. S1. Boxplots of main histological areas.**
(a) Thymic tissue is more prevalent in EOMG than LOMG but less than in pediatric samples. Area of fat, stroma and thymic tissue are shown as percentages of all tissue per sample. Thymic tissue is shown as the ratio to combined area of stroma and fat. Medulla is shown as the ratio to cortex. (b-c) Main histological areas or their ratios did not correlate with the outcomes after thymectomy at two years (b) or at the last follow up visit (c). (d) Histological profile of EOMG thymus is different from pediatric thymus.  P-values were calculated using the Kruskal-Wallis test.

# Supplemental Figure 2

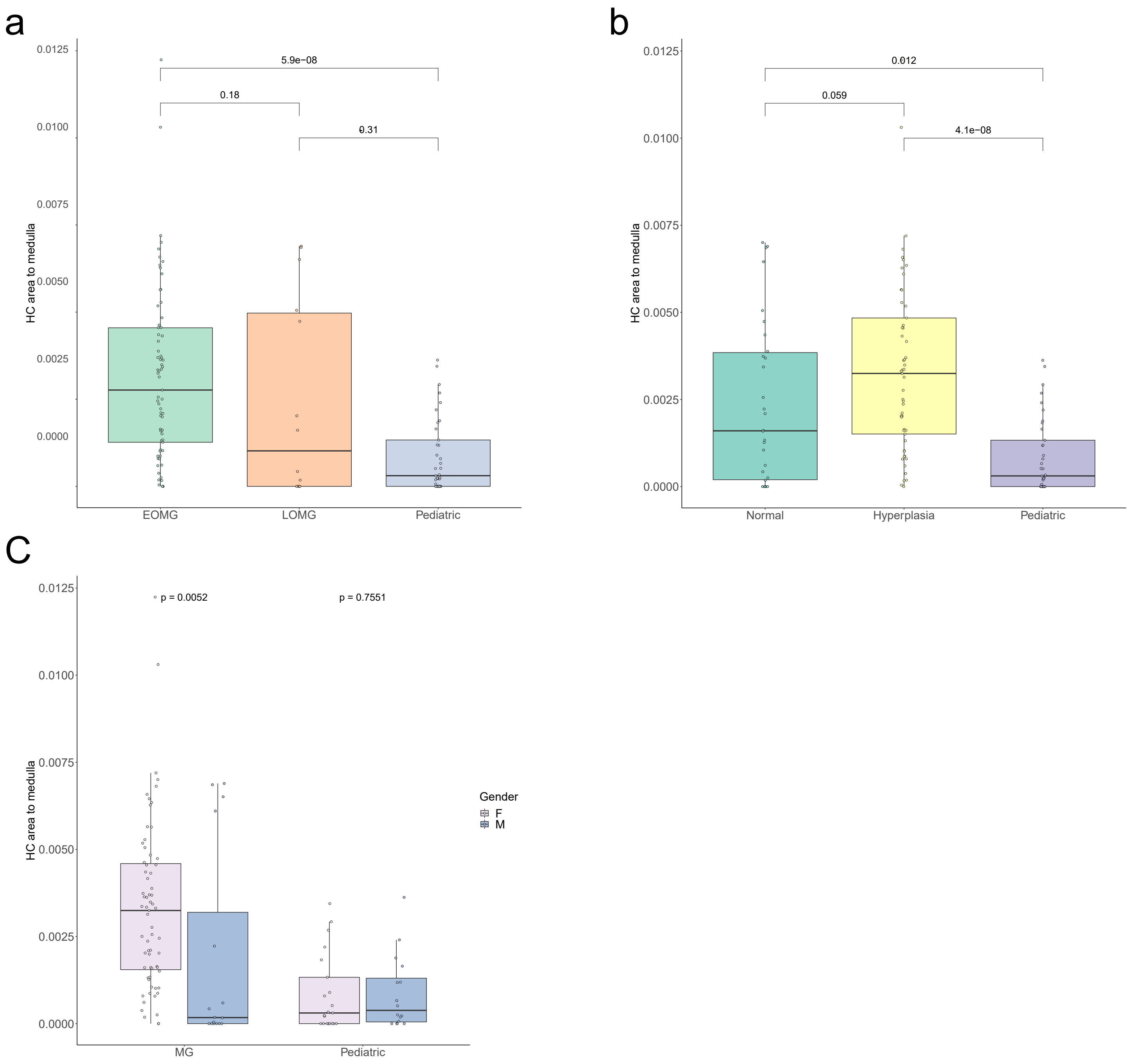


**Fig. S2. Comparison of area of Hassall Corpuscles (HCs) between EOMG, LOMG and healthy pediatric thymi.**
(a) Area of HCs is larger in EOMG thymi compared to healthy pediatric thymi. P-values were calculated using the Kruskal-Wallis test. (b) MG patients with thymic hyperplasia have a larger area of HCs in the thymus. P-values were calculated using the Kruskal-Wallis test. Area of HCs is normalized to the area of medulla in all the data presented here. (c) Female patients with MG show an increased area of HCs compared to male patients and pediatric controls. F, females; M, males. P-values were calculated using the unpaired Wilcoxon test.

# Supplemental Figure 3

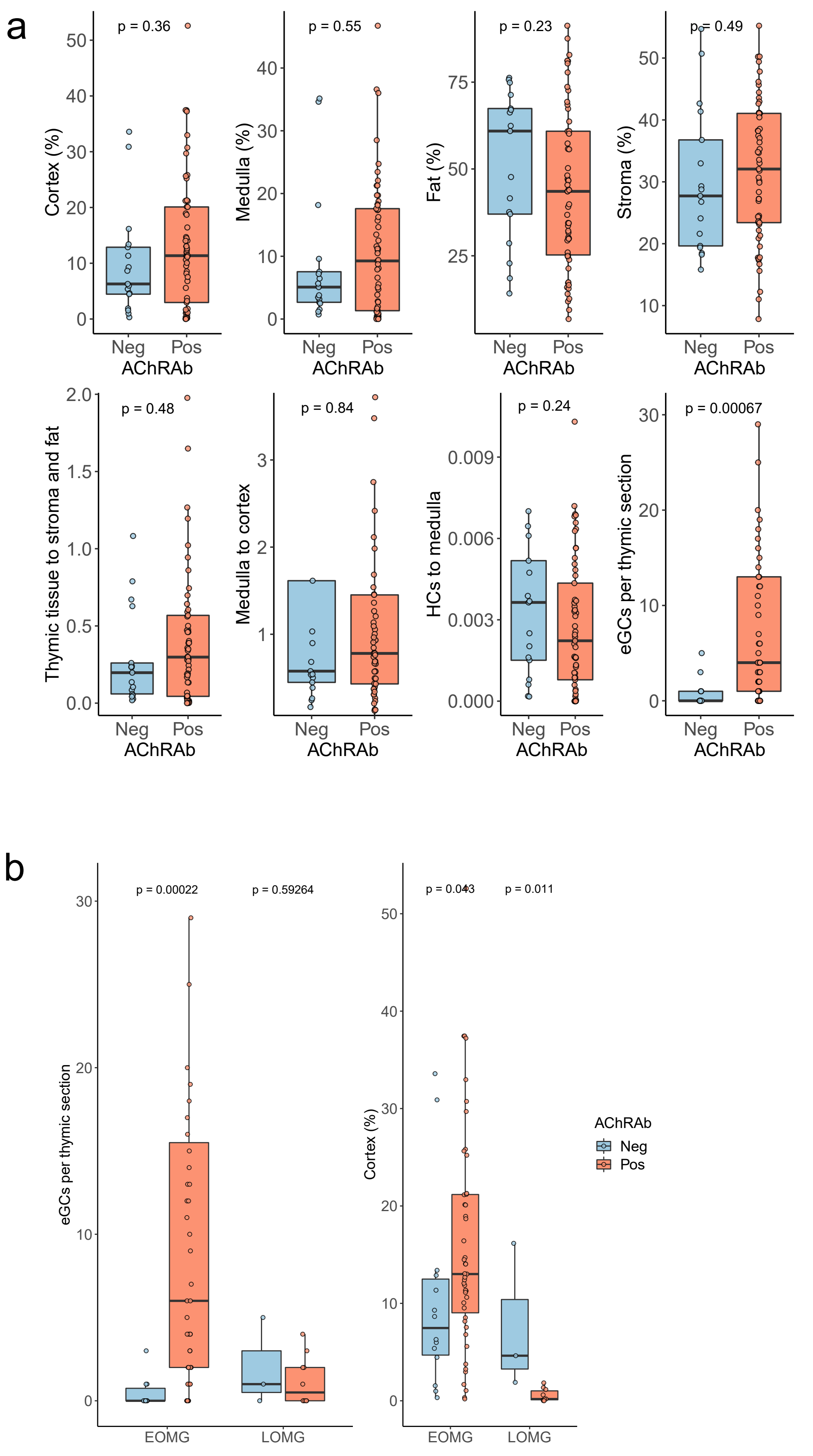


**Fig. S3. Boxplots of AChRAb status and main histological areas.**
(a) AChRAb seropositivity is correlated with increased amount of eGCs but not with other histological changes in the whole MG sample set. (b) Subgroup analysis between EOMG and LOMG shows that eGCs and area of cortex are increased in AChRAb seropositive EOMG patients but not in LOMG patients. P-values were calculated using the unpaired Wilcoxon test.

# Supplemental Figure 4

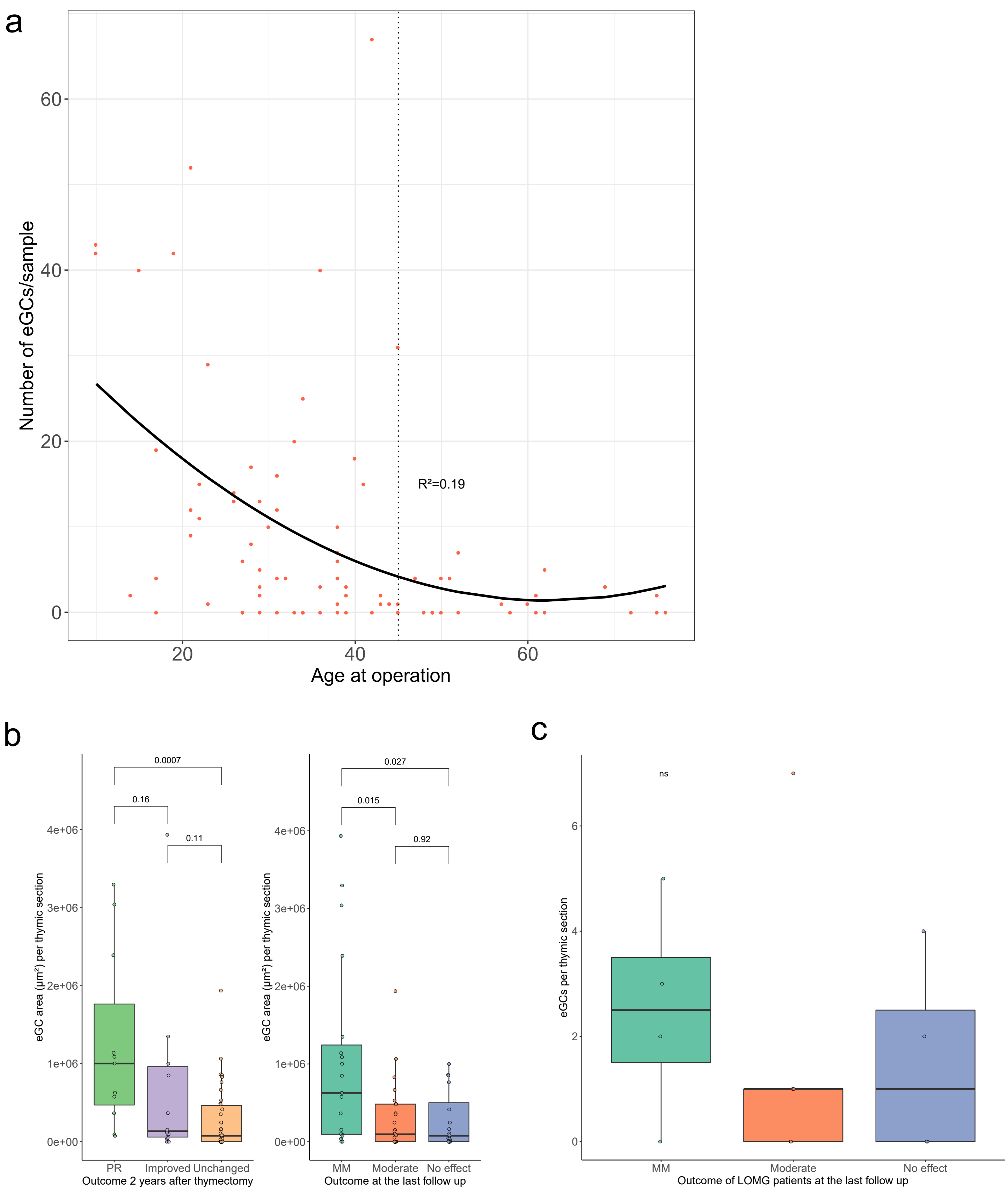


**Fig. S4. eGCs are more common in young MG patients and their prevalence is correlated with better outcome after thymectomy.**
(a) The number of eGCs per thymic slide in MG is inversely correlated with age and resembles a logarithmic curve. R-value is reported as adjusted R-squared value. (b) EOMG patients who benefitted the most from thymectomy had larger thymic eGC area. (i) Outcome two years after thymectomy. PR, pharmacological remission; Improved, -50% reduction in AChEi dose or no immunosuppressants in use; Unchanged, no change compared to preoperative state. (ii) Outcome at the last follow-up visit. MM, minimal need of medication; Moderate, AChEi dose over 100 mg/day or use of immunosuppressants; No effect, myasthenic crisis within one year. (c) No correlation between eGCs and outcomes were observed in LOMG. P-values were calculated using the Kruskal-Wallis test.
